# The eye of the beholder: orbital metastases from midgut neuroendocrine tumors, a two institution experience

**DOI:** 10.1186/s40644-018-0181-5

**Published:** 2018-12-06

**Authors:** Satya Das, Gino Pineda, Laura Goff, Rachel Sobel, Jordan Berlin, George Fisher

**Affiliations:** 1 Department of Internal Medicine, Vanderbilt University Medical Center, 1211 Medical Center Drive, Nashville, TN 37232 USA; 2 Department of Internal Medicine, Stanford University Medical Center, Stanford, CA USA; 3 Department of Opthalmology, Vanderbilt University Medical Center, Nashville, TN USA

**Keywords:** Small intestinal neuroendocrine tumors, Orbital metastases, 68Ga-DOTATATE PET-CT, Orbital MRI

## Abstract

**Background:**

Metastases to the orbit occur rarely in midgut neuroendocrine tumor (NET) patients with only 20 cases reported to date. Patients typically present with bilateral involvement of the recti muscles and experience symptoms such as diplopia, proptosis, and decreased vision. Although orbital MRI remains the gold standard for imaging orbital disease, many orbital lesions are now detected on somatostatin-receptor (SSTR) based imaging such as 68Ga-DOTATATE PET-CT.

**Case presentations:**

Patient 1 is a 72 year-old female with a well-differentiated G3 ileal NET who was incidentally diagnosed with orbital metastases during a hospitalization for pre-septal cellulitis in 2018. Her disease has been controlled with capecitabine rather than local therapy.

Patient 2 is a 68 year-old male with a G2 ileal NET who was diagnosed with orbital involvement after developing left peri-orbital swelling in 2017. He was found to have bilateral rectus muscle involvement and was treated with image-guided radiation therapy (IGRT) to both orbits and achieved disease control.

Patient 3 is a 63 year-old female with a well-differentiated G3 ileal NET who was incidentally diagnosed with bilateral orbital masses in her recti after undergoing a 68Ga-DOTATATE PET-CT in 2015. She was asymptomatic initially however has now developed diplopia. She will be starting 177Lu-DOTATATE peptide radionuclide receptor therapy (PRRT) shortly.

Patient 4 is a 72 year-old male with a grade 2 ileal NET who was incidentally diagnosed with a left lateral rectus metastasis in 2007. This was monitored via surveillance MRI until it began to grow and became symptomatic in 2015. The patient received stereotactic radiation to the site and has been asymptomatic since.

Patient 5 is a 61 year-old female with a grade 2 ileal NET who developed progressive diplopia in 2016. Bilateral orbital metastases were noted on orbital MRI and she completed IGRT to the sites shortly thereafter. In the setting of continued growth of the masses she was switched to chemotherapy with capecitabine which has controlled her orbital disease.

**Conclusions:**

NETs can metastasize to the orbits. Orbital disease now often is detected on SSTR-based imaging rather than orbital MRI; when found, it changes treatment approach and surveillance for patients.

## Background

Orbital metastases represent an exceedingly rare metastatic manifestation of neuroendocrine tumors (NETs) and occur through hematogenous dissemination via the carotid and ophthalmic artery [1]. Orbital involvement, specifically from NETs of midgut primary origin, represents an even less common occurrence with our literature review identifying 20 such published cases [1–6]. Intraocular metastases from midgut NET patients, specifically to the uvea, may be more frequent than midgut NET orbital metastases [2].

The most common symptoms at presentation for patients with metastatic orbital involvement include diplopia, proptosis, and decreased vision. Other common symptoms include eyelid swelling, chemosis and redness. Unilateral rather than bilateral involvement is more frequent, and the rectus muscles are often involved [7, 8]. The gold standard for imaging orbital metastases remains an orbital MRI; however, as sensitivity of systemic imaging modalities for NETs have improved, this is not always how lesions are first detected now. Specifically, improvements in somatostatin receptor (SSTR)-based imaging and widespread adoption of 68Ga-DOTATATE PET-CT has resulted in the detection of more patients with occult metastatic lesions [9, 10]. On cross-sectional imaging, NET metastases to the orbit tend to be well circumscribed and mass-like rather than infiltrative or inflammatory [11, 12].

Patients with orbital involvement from NETs typically carry an excellent prognosis with 10-year survival rates of close to 40%, which is in stark contrast to the poor systemic prognosis seen in other malignancies when orbital involvement is detected [13]. This makes local control even more important, particularly for symptomatic patients. Some common treatments for patients with orbital metastases include surgical debulking, radiation therapy and systemic treatment (somatostatin analogs, 177Lu-DOTATATE peptide radionuclide receptor therapy (PRRT), everolimus and others). In this series, we describe the experience of 5 midgut NET patients with extra-ocular orbital metastases from Stanford University Medical Center (SUMC) and Vanderbilt University Medical Center (VUMC) and explore management strategies for patients with orbital disease published in existing literature.

## Case presentations

### Patient 1

**Fig. 1:**
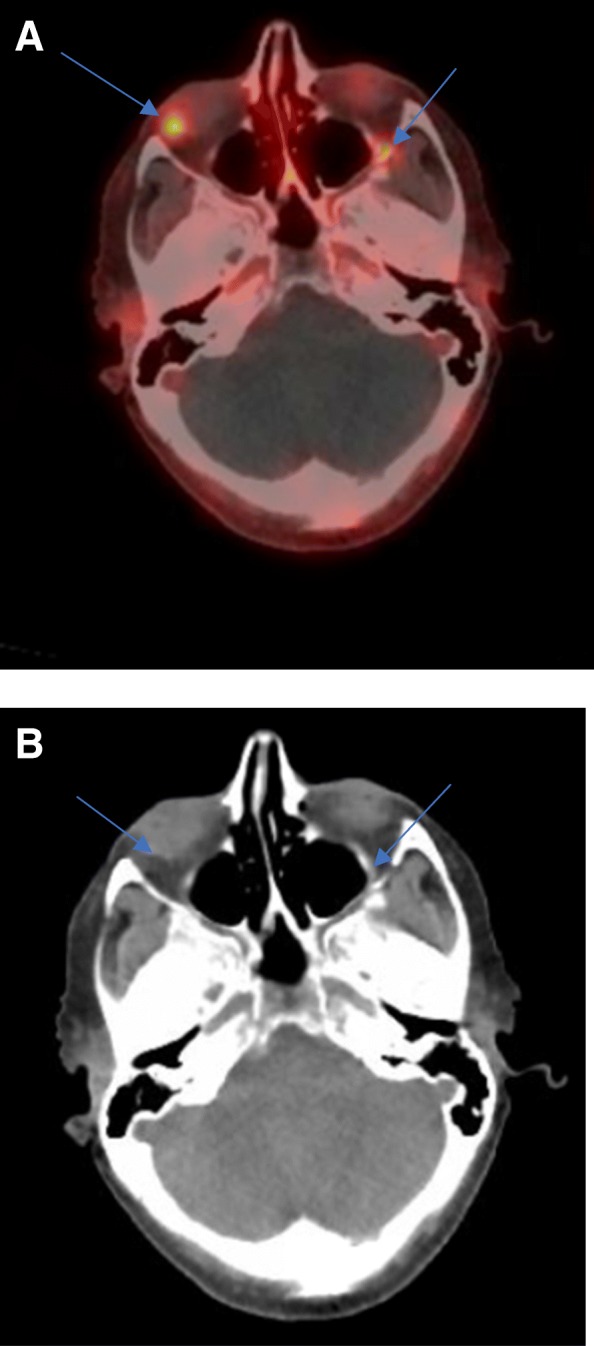
(**a**) 68Ga- DOTATATE PET/CT (2017) from patient 1. SSTR avidity is demonstrated in bilateral orbits in A and is indicated by blue arrows. Blue arrows on the CT, image (**b**), represent the corresponding metastatic lesions. The left orbital lesion is more difficult to visualize on the CT

Patient 1 is a 72-year-old female who was initially diagnosed in 1997 with a well differentiated G3 (Ki-67 20%) metastatic jejunal NET. Her primary tumor was resected after diagnosis and a liver transplant was performed in 1998 for bulky symptomatic liver metastases. She has received a series of therapies including octreotide, lanreotide, and everolimus for her carcinoid syndrome (CS) and disease control. In May 2017, a 68Ga-DOTATATE PET-CT revealed somatostatin receptor (SSTR) avidity in bilateral orbits although she was asymptomatic from a visual symptom standpoint (Fig. 1a and b). In March 2018, the patient was hospitalized for preseptal cellulitis. During the hospitalization, she underwent a CT scan which revealed focal thickening of the right medial rectus and left lateral rectus muscles. Opthalmology evaluated her and felt the cellulitis was unrelated to her orbital masses. Once her infection resolved she underwent biopsy of a rectus muscles mass which confirmed orbital metastases from her NET primary. Patient did not have any worsening visual symptoms or signs (limited extraocular motility, visual field deficits or proptosis).

177Lu-DOTATATE PRRT was initially recommended; however, this was not pursued due to the patient’s poor renal function. Radiation therapy was then considered but given the proximity of the lesions to other critical structures and her lack of symptoms, this too was deferred in favor of close surveillance. In July 2018, she started on capecitabine and temozolomide for better systemic control of her NET, which also involved her liver, pancreas, bone and intra-abdominal lymph nodes. On a follow-up orbital MRI in July 2018, her lesions were noted to be stable.

### Patient 2

**Fig. 2:**
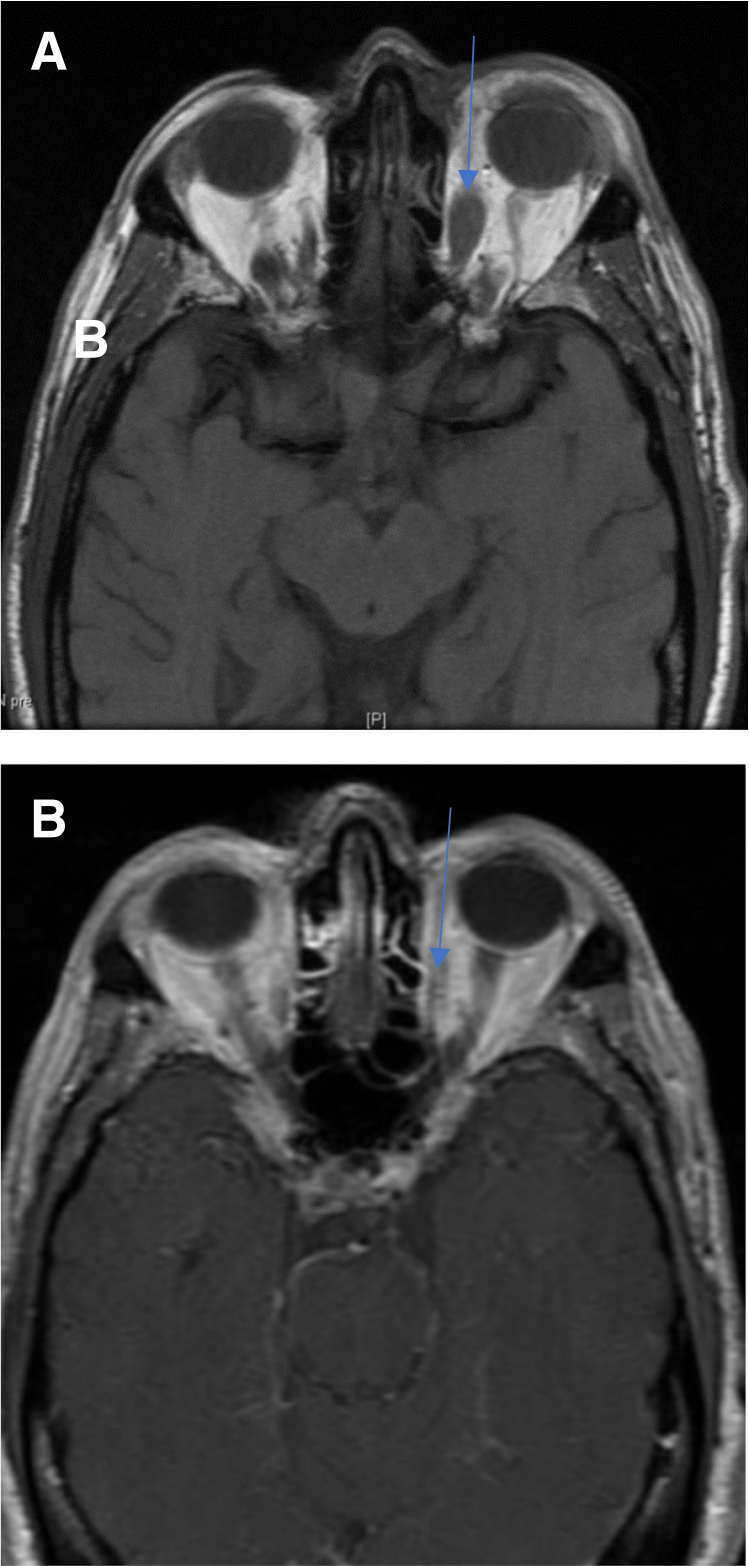
Pre (A, 2017)- and post (B, 2018)-radiation T1 weighted orbital MRI studies from patient 2. The blue arrow in (**a**) points to his left medial rectus mass pre-treatment and in (**b**), his post-treatment mass which demonstrates reduced enhancement

Patient 2 is a 68-year-old male who was initially diagnosed with a well-differentiated G2 (Ki-67 10%) metastatic ileal NET in 2012. Post-resection of his primary, the patient began treatment with octreotide. Eventually, he enrolled on a clinical trial with 177Lu-DOTATATE PRRT. During a hospitalization in November 2017, while admitted for abdominal pain, he developed left peri-orbital swelling. This prompted an orbital MRI which demonstrated bilateral extraocular masses in his recti muscles (Fig. 2a). A subsequent biopsy confirmed metastatic NET. To manage his acute periorbital swelling, he was first treated with corticosteroids and later completed image-guided radiation therapy (IGRT) to 44 Gy to bilateral orbits in December 2017. The patient had a post-treatment MRI scan which demonstrated a decrease in signal abnormality and enhancement in the previously visualized lesions (Fig. 2b). Post-radiation, patient continued octreotide until his death in February 2018 due to progressive disease.

### Patient 3

**Fig. 3:**
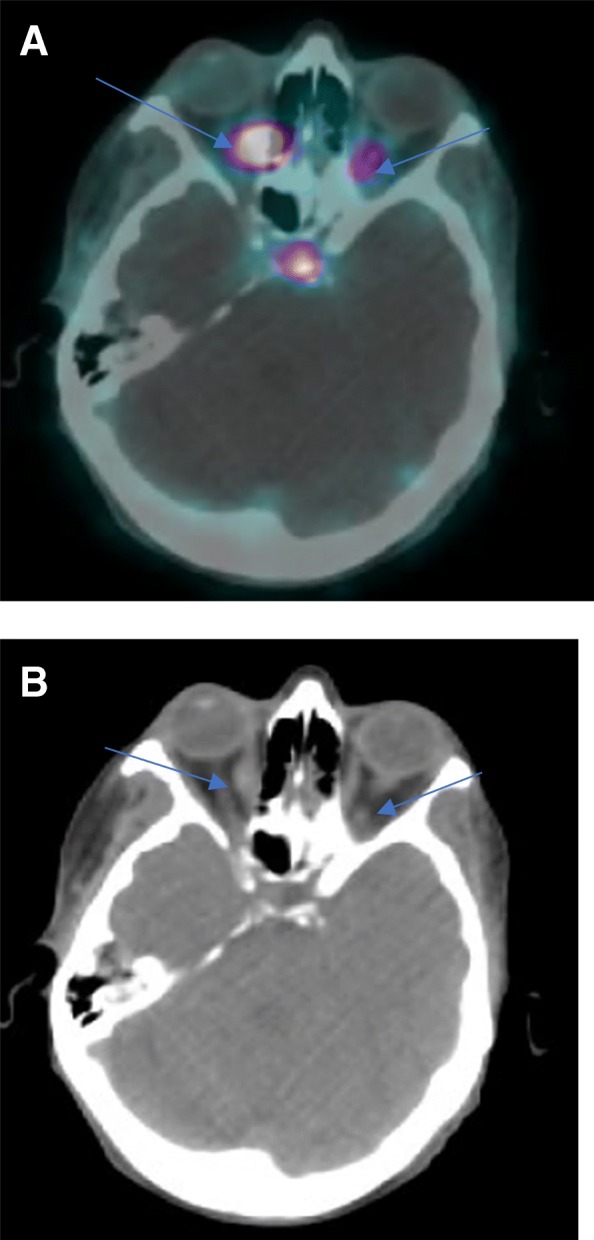
The most recent 68Ga-DOTATATE PET/CT scan (2018) from patient 3. The blue arrows in (**a**) correspond to SSTR avidity in her right medial and left infraorbital recti muscles while the blue arrows in B indicate the mass lesions on the corresponding CT

Patient 3 is a 63-year-old female who was initially diagnosed with a well differentiated metastatic ileal NET in 1994. She was found to have G3 (Ki-67 22%) disease on later biopsies. She had stable disease controlled on octreotide until 2014 when she had disease progression which required everolimus, multiple hepatic artery embolizations and debulking of bulky adenopathy in her right paratracheal region. She underwent her first 68Ga-DOTATATE PET-CT in April 2015 which revealed bilateral extraocular masses in her right medial and left infraorbital rectus muscles. She was asymptomatic initially from these lesions; however, during her initial visit at VUMC in June 2018 for consideration of 177Lu-DOTATATE PRRT, due to persistent CS, she mentioned worsening diplopia and visual acuity. A pre-treatment 68Ga-DOTATATE PET-CT was repeated which revealed increased SSTR avidity in the recti at the site of the previously known lesions (Fig. 3a and b). Although she was planned for earlier, in the setting of disease-related complications, her anticipated start date for 177Lu-DOTATATE PRRT is December 2018.

### Patient 4

**Fig. 4:**
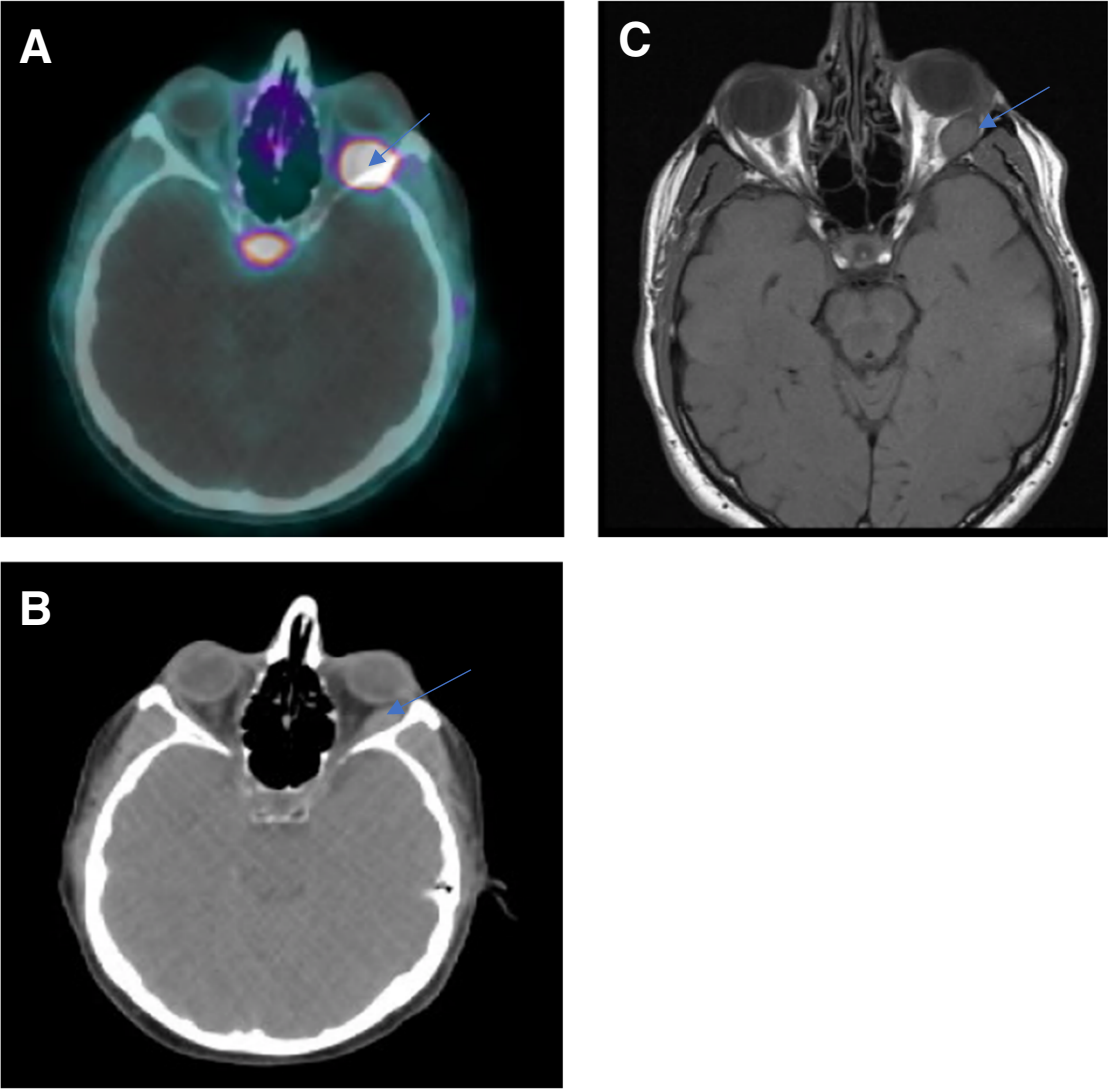
The most recent 68Ga-DOTATATE PET CT (2018) from patient 4. The blue arrow in (**a**) points to a focus of SSTR avidity in the left lateral rectus muscle while in (**b**) points to the corresponding CT mass lesion. The arrow in image (**c**), a T1 weighted MRI image (2015), points to the left lateral rectus mass at its largest dimension

Patient 4 is a 72-year-old gentleman who was diagnosed with a well differentiated G2 metastatic ileal NET in 2006. He started monthly octreotide shortly thereafter in 2007. He was incidentally noted to have a left lateral rectus mass in March 2007 during a brain MRI. This lesion was monitored with serial MRI and remained stable until 2015 when it began to grow (Fig. 4c). The patient developed progressive diplopia and proptosis in 2015 which prompted an evaluation by radiation oncology. He received stereotactic radiosurgery (SRS) administered over 5 fractions to the site in February 2015 at VUMC and his diplopia and proptosis resolved within several months of treatment completion. He has unfortunately developed complications from his other sites of metastatic involvement including right sided nephrostomy tube placement from ureteral obstruction and an end colostomy due to recurrent small bowel obstructions from mesenteric tethering. His disease remains radiographically stable on his 68Ga-DOTATATE PET-CT from August 2018 (Fig. 4a and b).

### Patient 5

**Fig. 5:**
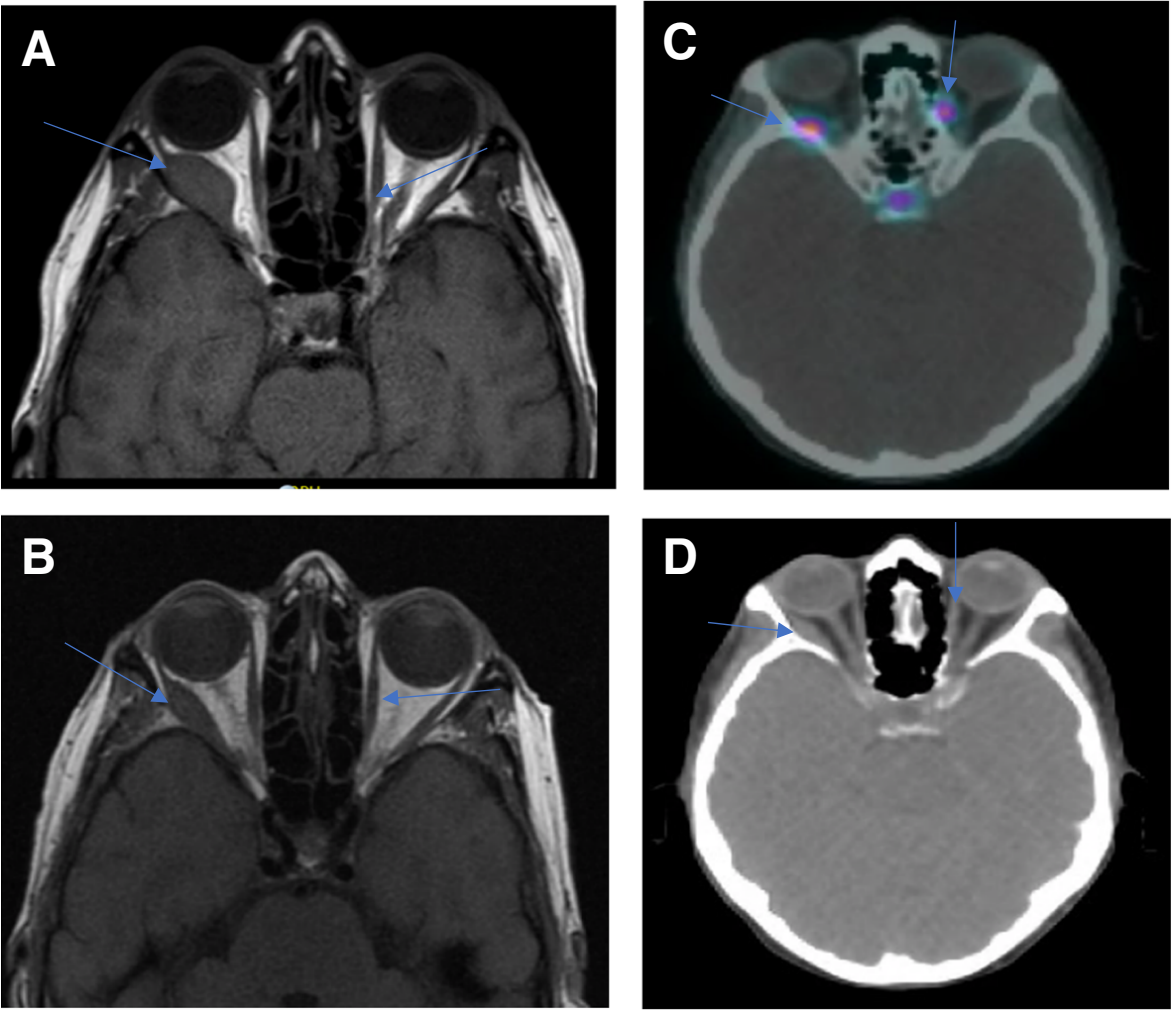
T1 weighted orbital MRI scans of patient 5 pre (2017)- and post-18 months of capecitabine (2018) and her most recent 68Ga-DOTATATE PET CT (2018). Arrows in (**a**) correspond to the bilateral recti masses pre-capecitabine while arrows in (**b**) correspond to those same masses after patient initiated capecitabine. Arrows in (**c**) highlight the SSTR avid lesions in bilateral recti while in (**d**) point to the masses on the corresponding CT

Patient 5 is a 61-year-old female who was diagnosed with a well differentiated G2 (Ki-67 5%) metastatic small intestine (not otherwise specified) NET in February 2010. She remained symptom free until April 2014 when she was started on monthly octreotide. In the setting of progressive disease, she subsequently received sunitinib and everolimus. She began to develop progressive diplopia and right ocular pain in August 2016. This prompted an orbital MRI which revealed bilateral recti masses in the right lateral and superior left medial muscles. She was evaluated by radiation oncology shortly thereafter and was treated with IGRT to 52Gy, which completed in October 2016. Although her right ocular pain improved, she had persistent diplopia. Her post-treatment orbital MRI in January 2017 revealed a mild increase in size of her right lateral rectus mass (Fig. 5a). She was then started on capecitabine in January 2017 on a two week on, one week off regimen schedule. Patient also established care with ophthalmology at this time. She achieved stable disease in her orbits with symptomatic improvement and did not demonstrate any evidence of visual field deficits. She continues capecitabine and her last MRI in July 2018 revealed ongoing shrinkage of her right lateral rectus mass (Fig. 5b); her recent 68Ga-DOTATATE PET/CT from October 2018 shows residual SSTR avidity within her bilateral recti muscles (Fig. 5c and d).

## Discussion

We have described here five cases of midgut NET patients who presented with orbital metastases at various stages of their disease presentation. Of the five patients, three were initially asymptomatic from their orbital involvement. Four out of the five patients presented with bilateral involvement, which is different from the unilateral predominant pattern described in the literature. This discrepancy may be due to the increased sensitivity of current imaging modalities which capture lesions missed by prior imaging techniques. All the patients had rectus muscle involvement which is the most common orbital structure involved from existing case descriptions (Table 1). Orbital involvement suggests significant hematogenous dissemination and typically occurs in the context of other metastatic involvement [7]. Consistent with this observation, all five of the patients in our series had more than two sites of other metastatic involvement, with the most common ones being liver (4), bone (4), pancreas (3) and mesentery (3). Only one of five patients underwent an orbital biopsy to establish the diagnosis. While the gold standard in the past used to be orbital biopsy to confirm diagnosis [13], we believe in patients with a history of midgut NET with other systemic metastases and with classic radiologic findings, empiric therapy can proceed without biopsy.

**Table 1:** Characteristics of the five patients in our series. R = right and L = left. SRS = stereotactic radiosurgery and IGRT = image guided radiation therapy

Patient (age, gender)	Primary Site	Grade	Ki-67% (highest if multiple lesions biopsied)	Unilateral or Bilateral Orbital Involvement (Specific Recti Involved)	Ocular Symptoms	Other Metastatic Sites	Local Treatment Received for Orbital Involvement
1 (72, female)	Midgut (Jejunum)	G3	>20%	Bilateral (R medial rectus, L medial rectus, L lateral rectus)	None	Liver, pancreas, intra-abdominal lymph nodes, bone	No radiation
2 (68, male)	Midgut (Ileum)	G2	10%	Bilateral (L extraocular muscles except lateral rectus, R inferior rectus, R inferior oblique, R superior rectus, R medial rectus)	L periorbital swelling and pain	Liver, bone	IGRT 11/2017
3 (63, female)	Midgut (Ileum)	G3	22%	Bilateral (R medial, L infraorbital)	Diplopia, diminished vision	Calvarium, axial spine, thoracic nodes, liver, pancreas, peritoneum, pericardium	No radiation
4 (72, male)	Midgut (Ileum)	G2	NA	Unilateral (L lateral rectus)	Proptosis, diplopia	Mesentery, pelvis, pancreas, omentum	SRS 2/2015
5 (61, female)	Midgut (NOS)	G2	<10%	Bilateral (R lateral rectus, L superior oblique)	Diplopia	Mesentery, bone, liver, mediastinal lymph nodes	IGRT 10/2016

Three of the five patients were treated with external beam radiation; two of these patients received IGRT and one received five fractions of stereotactic radiosurgery. Two of the three patients who received radiation achieved improved visual symptoms and disease control locally. One patient progressed through IGRT, but eventually achieved disease control with capecitabine. Of the two patients who did not receive radiation therapy, one achieved intra-orbital disease control with capecitabine plus temozolomide. We are hopeful the other patient will also achieve orbital disease control with PRRT. None of the patients in our series had any attempt at surgical resection.

Prior to the 1980s, orbital exenteration was considered the recommended treatment for orbital metastases [13]. With improvements in non-surgical therapies, the role of surgery, even debulking, due to the morbidity of diplopia and vision loss is minimal. From other published series and our own experience, the current first line option for symptomatic patients appears to be IGRT alone [1]. Many of the treatment planning fields utilized for IGRT to the orbits are similar to fields utilized for patients with Graves opthalmopathy [14]. In patients who are not radiation candidates due to the proximity of their orbital masses to other critical structures, systemic chemotherapy and targeted therapy are other options. In patients who fail radiation therapy, such as our fifth patient, these other options can also be explored.

One of the therapeutic areas of great promise in midgut NET is PRRT. 177Lu-DOTATATE PRRT was FDA approved in January 2018 and represents another cytoreductive treatment option available for patients. In Europe, PRRT approaches with other radionuclides such as 90Y have been utilized successfully. 177Lu-DOTATATE PRRT involves targeted radiation where lutetium 177, a beta emitting radionuclide, is conjugated to a SSTR analog and is internalized by neuroendocrine cells expressing SSTR2 [15]. Reports demonstrate overall response rates of 17–38% with this therapy which is markedly higher than many of the other approved cytostatic systemic therapies for NET patients [16, 17]. There have been several published case reports documenting symptomatic relief and stable radiographic findings after 177Lu-DOTATATE PRRT treatment in patients with symptomatic metastases from orbital involvement [18–20].

## Conclusions

Orbital metastases are rare, occurring in 2–3% of all malignancies [7]. Of the orbital metastases that have been reported, the majority originate from more common malignancies such as breast cancer, lung cancer, prostate cancer and melanoma [11]. NETs metastasize to the orbits in 4–5% of patients despite representing a much rarer subgroup. In our series, specifically of midgut NETs metastatic to the orbit, we demonstrated that surgical biopsy is not necessary for diagnosis in the setting of a previously established diagnosis, systemic metastases and classic radiologic findings. This series also shows that radiation (via IGRT) rather than exenteration or debulking, with adjunctive support from chemotherapy, is an effective treatment with minimal morbidity. The importance of multidisciplinary care in the management of these patients cannot be underestimated. Early referral to ophthalmology to gauge baseline visual function and discussions amongst radiation oncology, surgical oncology and medical oncology to develop a treatment plan prioritizing quality of life (in the palliative setting) are necessities for each patient. How PRRT changes the treatment landscape for patients with orbital involvement, specifically in relation to utilization of local radiation, remains to be determined.

## Abbreviations

IGRT: Image-guided radiation therapy
NETs: Neuroendocrine tumors
PRRT: Peptide radionuclide receptor therapy
SSTR: Somatostatin receptor
